# Can cytology accurately predict the malignancy of feline mammary gland tumours?

**DOI:** 10.18849/ve.v11i2.733

**Published:** 2026-05-22

**Authors:** Juulia Virtanen+

**Keywords:** CYTOLOGY, FELINE, FINE NEEDLE ASPIRATION, HISTOPATHOLOGY, MAMMARY CARCINOMA, MAMMARY TUMOUR

## Abstract

**PICO question:**

In cats with mammary gland tumours, is cytology compared to histopathology an accurate method for predicting the malignancy of the tumour?

**Clinical bottom line:**

**Category of research:**

Diagnosis.

**Number and type of study designs reviewed:**

One case series and two diagnostic accuracy studies.

**Strength of evidence:**

Moderate.

**Outcomes reported:**

In cats with mammary gland tumours, cytology can reliably predict the malignancy of the tumour when compared to histopathology as the clinical reference standard.

**Conclusion:**

There is moderate evidence to show that the diagnostic accuracy of cytology in predicting the malignancy of feline mammary gland tumours is high, especially in terms of sensitivity, but further research is needed to confirm its specificity. Due to the current uncertainties, all preoperative cytological diagnoses should be confirmed with a postoperative histopathological examination.

How to apply this evidence in practice

The application of evidence into practice should take into account multiple factors, not limited to: individual clinical expertise, patient’s circumstances and owners’ values, country, location or clinic where you work, the individual case in front of you, the availability of therapies and resources.

Knowledge Summaries are a resource to help reinforce or inform decision making. They do not override the responsibility or judgement of the practitioner to do what is best for the animal in their care.

## Clinical scenario

A 16-year-old female neutered domestic shorthair cat, that has previously been diagnosed with hypertrophic cardiomyopathy and chronic kidney disease, presents for further evaluation of a small nodule in the right inguinal area. The nodule had been discovered by the owner two weeks earlier. No apparent growth has been noted during this time, and the patient does not seem to be bothered by it. Physical examination confirms the presence of a firm 1.5 cm nodule in the right fourth mammary gland. The ipsilateral inguinal lymph node cannot be palpated.

The differential diagnoses and treatment options, including radical mastectomy, are discussed with the owner. Considering the patient’s advanced age and comorbidities, the owner expresses concern about the extensiveness of radical mastectomy and asks if there is a reliable method to ascertain the malignancy of the mass and thereby the required treatment protocol preoperatively.

## The evidence

The literature search returned three studies that were applicable to the PICO question: one case series (Amorim et al., 2006) that investigated the diagnostic accuracy of cytology compared to histopathology while describing the clinical, cytological and histopathological characteristics of a local population of cats with mammary gland tumours; and two diagnostic accuracy studies (Pakdeesaneha et al., 2024; and Simeonov, 2024) that specifically aimed to compare the diagnostic accuracy of cytology to histopathology as the clinical reference standard. In the hierarchy of evidence, the results of the case series represent a low level of evidence, whereas those of the diagnostic accuracy studies represent a high level of evidence. Collectively, these three studies provide moderate evidence to support the diagnostic accuracy of cytology in predicting the malignancy of feline mammary gland tumours, especially in terms of sensitivity. In addition, one of the studies (Pakdeesaneha et al., 2024) provides weak evidence to suggest that, in terms of diagnostic accuracy, core-needle biopsies are a nonsuperior method compared to fine-needle aspiration biopsies.

## Summary of the evidence

**Amorim et al. (2006):** Clinical, cytological and 198 histopathological evaluation of mammary masses in cats from Rio de Janeiro, Brazil **Aim: **To evaluate 20 cats with mammary gland masses using clinical findings, cytology and histopathology.

Population:	Cats with mammary masses being evaluated at veterinary hospitals in Rio de Janeiro, Brazil between February 2002 and December 2004.
Sample size:	20 cats.
Intervention details:	Cytology (15 cats): Fine-needle aspiration biopsies were performed on 15 mammary gland masses. The owners of 5/20 cats declined cytology. Staining was performed with Papanicolaou stain. Histopathology (20 cats): Biopsy samples were collected with incisional biopsy in 1 cat and with excisional biopsy or necropsy in 19 cats. Evaluation of the complete unilateral mammary chain was performed in 10 cats. The biopsies were fixed in 10% formalin, trimmed, processed in an automatic tissue processor, sectioned in 6-µm slices and stained with haematoxylin and eosin.
Study design:	Prospective, multicentre, case series.
Outcome Studied:	Subjective assessment of the cytological and histopathological features of the tumours and the agreement between the cytological and histopathological diagnosis. Multiple additional outcomes not relevant to the PICO question.
Main Findings (relevant to PICO question):	All of the included tumours (n = 15, 100%) were malignant. Cytological diagnosis was in agreement with the histopathological diagnosis in 15/15 cases (100%).
Limitations:	The study type (prospective, multicentre, case series) is not stated by the authors of the article but was determined by the author of this Knowledge Summary. Case series are located at the bottom of the hierarchy of evidence as the lack of a control group makes them prone to selection bias and reduces their internal and external validity. The sample size is small which reduces the reliability and generalisability of the results as random variation can have a major effect on the collected data. No inclusion or exclusion criteria are reported, and geographical and referral bias may be present as all the samples were collected at veterinary hospitals in one city. These factors further limit the generalisability of the results. The sampling technique (e.g. the needle and syringe sizes and whether aspiration was used), sample preparation technique (e.g. the fixation method) and the number and experience of the investigators preparing and evaluating the samples are not reported even though these factors may impact the reliability of the results, and their absence reduces the reproducibility of the study. The number of cytological malignancy criteria required to be met for classifying the tumour as malignant is not reported. The sensitivity, specificity, positive predictive value or negative predictive value of cytology are not reported. Contradictorily, as only malignant tumours were included, no true conclusions on these aspects could even be drawn.

**Pakdeesaneha et al. (2024):** Comparison of Fine-Needle Aspiration and Core Needle Biopsy for the Pre-Operative Diagnosis of Canine and Feline Mammary Gland Tumours **Aim: **To compare the diagnostic accuracy of fine-needle aspiration biopsies and core-needle aspiration biopsies to excisional tissue histopathology in the diagnosis of feline mammary gland tumours.

Population:	Cats with mammary gland masses undergoing mastectomy at the Small Animal Teaching Hospital of Chulalongkorn University (Bangkok, Thailand) between January 2022–December 2022.
Sample size:	64 cats.
Intervention details:	All cats (n = 64) underwent fine-needle aspiration (FNA) biopsies and excisional mammary tissue biopsies. All cats (n = 64) were randomised (technique not specified) to undergo core-needle biopsies (CNB) with either an 18-gauge (n = 20) or 16-gauge (n = 44) needle. Cytology (64 cats): Sampling was performed preoperatively under general anaesthesia by a single animal reproduction specialist. In the case of multiple masses, only the largest was sampled. FNA: sampling was performed with a 24-gauge needle using aspiration technique. The obtained material was smeared onto glass slides, fixed by air-drying and stained. CNB: sampling was performed with an 18-gauge or 16-gauge needle attached to a spring-loaded gun. The obtained material was fixed in 10% formalin. Cytology was evaluated by a blinded pathologist. Robinson’s grading system was used to assess malignancy. Histopathology (64 cats): A small (1 x 1 x 1 cm) excisional biopsy of each cytologically evaluated mammary gland tumour was collected postoperatively. The biopsies were fixed in formalin, embedded in paraffin, sectioned and stained. Mills grading system was used to confirm malignancy. Histopathology was evaluated by a blinded certified veterinary pathologist.
Study design:	Prospective, randomised, blinded, diagnostic, accuracy study.
Outcome Studied:	Subjective assessment of the cytological and histopathological features of the tumours using objective assessment criteria (Robinson’s grading system for cytology and Mills scoring system for histopathology). Objective assessment of the agreement between the cytological and histopathological diagnosis of the mammary gland tumours.
Main Findings (relevant to PICO question):	Of the 20 cats undergoing FNA and CNB with an 18- gauge biopsy needle, results agreed with the histopathological diagnosis in 19/20 (95%) of the tumours, and all the correctly diagnosed tumours (19/19, 100%) were malignant. CNB results agreed with the histopathological diagnosis in 18/20 (90%) of the tumours, and all the correctly diagnosed tumours (18/18, 100%) were malignant. No significant difference in the diagnostic accuracy of FNA or CNB was found (P = 1.0). Of the 44 cats undergoing FNA and CNB with a 16- gauge biopsy needle, FNA results agreed with the histopathological diagnosis in 40/44 (90.9%) of the tumours, and 39/40 (97.5%) of them were malignant. CNB results agreed with the histopathological diagnosis in 43/44 (97.7%) of the tumours, and all the correctly diagnosed tumours (43/43, 100%) were malignant. No significant difference in the diagnostic accuracy of FNA or CNB was found (P > 0.05).
Limitations:	The sample size is relatively small and no sample size calculation is provided. This reduces the statistical power of the study and the reliability and generalisability of its results as random variation can have a major effect on the collected data. The study population is not described in enough detail (e.g. the ages, breeds, or sexes of the patients are not included), and geographical and referral bias may be present as all the samples were collected at a single referral clinic. These factors further limit the generalisability of the results. Selection bias may be present as including mastectomy patients only can lead to overrepresentation of malignant tumours. The randomisation method and the blinding process are not described in enough detail to allow critical assessment of the internal validity of the study. The FNA and CNB were obtained from patients under general anaesthesia which differs from common practice. This may improve the diagnostic quality of the samples compared to ones obtained from conscious or mildly sedated patients and thereby bias the results towards diagnostic accuracy of cytology. It also reduces the generalisability of the results. Only two or three cytological samples were collected from each tumour, and the distance between the cytological sampling site and the excisional biopsy site is not reported. These factors can introduce measurement bias and falsely reduce the reported diagnostic accuracy of cytology. Histopathology was performed on a small excisional biopsy instead of the entire surgically removed mammary gland tissue. This can introduce measurement bias and risk the diagnostic accuracy of the histopathological assessment used as the clinical reference standard. Whether a certified veterinary pathologist evaluated the cytological samples (similarly to the histopathological samples) is not clearly stated even though the importance of the pathologist’s experience is emphasised in the introduction and can have a major effect on the reliability of the results. Despite the study being a purposely designed diagnostic accuracy study, no calculation of the overall sensitivity, specificity, positive predictive value or negative predictive value of cytology is provided, nor are any confidence intervals included. If this is related to the low number of benign tumours, which considerably limits the reliability of these assessments (especially in terms of specificity and negative predictive value), it should have been clearly stated and discussed as an important limitation of the study.

**Simeonov (2024):** Correlation between fine-needle aspiration biopsy and routine 264 histopathology in the diagnosis of spontaneous feline mammary gland tumours **Aim: **To evaluate the correlation between fine-needle aspiration biopsies and histopathological examination in the diagnosis of feline mammary gland tumours.

Population:	Cats with mammary masses evaluated at the surgical clinic of the Faculty of Veterinary Medicine, Trakia University (Stara Zagora, Bulgaria) between the years 2000 and 2010.
Sample size:	120 feline mammary gland tumours.
Intervention details:	Cytology (120 mammary gland tumours): Fine-needle aspiration (FNA) biopsies were performed preoperatively on all mammary gland tumours with a 20- or 22-gauge needle and a 10 ml syringe. Aspiration technique was used and sampling of the centre of the tumour was avoided to reduce the risk of aspirating necrotic material. Fixation was performed with Merckofix spray and staining with Hemacolor. Criteria listed in a cytology book were used to assess malignancy. Histopathology (120 mammary gland tumours): Biopsies were sectioned in 4 µm slices and stained with haematoxylin and eosin. Criteria listed in pathology books were used to confirm malignancy.
Study design:	Non-blinded diagnostic accuracy study.
Outcome Studied:	Assessment of the cytological and histopathological features of the tumours using objective assessment criteria listed in cytology and pathology books. Assessment of the sensitivity and specificity of cytology compared to histopathology as the clinical reference standard.
Main Findings (relevant to PICO question):	102/120 (85%) of the tumours were malignant and 18/120 (15%) of the tumours were benign. Cytological diagnosis was in agreement with the histopathological diagnosis in 111/120 cases (92.5%) and incorrect in 9/120 cases (7.5%) with 3/120 (2.5%) being false positives and 6/120 (5%) false negatives. Among malignant tumours, cytological diagnosis was correct in 94/102 (92.2%) and incorrect in 8/102 (7.9%) of cases (false negative in 5/102 [4.9%] and false positive in 3/102 [2.9%] of cases). Among benign tumours (n = 18), cytological diagnosis was correct in 17/18 (94.4%) and false negative in 1/18 (5.6%) of cases. The overall sensitivity of cytology for diagnosing feline mammary gland tumours was 95.2%, specificity 75%, positive predictive value 97.5% and negative predictive value 60% when compared to histopathology as the clinical reference standard.
Limitations:	The number of actual patients is not reported, and there is no description of the study population or the inclusion and exclusion criteria. In addition, geographical bias may be present as all the samples were collected in Bulgaria. These factors limit the generalisability of the results. It is unclear whether the study was prospective or retrospective. The number of collected cytological samples, the active ingredients of the fixative and stain, the number and collection method of the histopathological samples and the number and experience of the investigators collecting and evaluating the samples are not reported, even though these factors may impact the reliability of the results, and their absence reduces the reproducibility of the study. The apparent lack of blinding can introduce performance bias as being aware of the results of either cytology or histopathology can guide the investigator’s interpretation of the other modality’s results and lead to a falsely high agreement between the two. Old editions of cytology and pathology books are stated as the source of the applied malignancy criteria instead of a specific grading system (e.g. the Elston and Ellis, or the Mills grading system) which may add more subjectivity to the malignancy assessment and reduce the reliability of the results and the reproducibility of the study. No confidence intervals to help assess the reliability of the reported results are provided.

### Appraisal, application and reflection

Mammary gland tumours are one of the most commonly occurring tumours in cats (Giugliano et al., 2024; and Srisawat et al., 2024). The vast majority of affected patients are 9- to 12-year-old females, even though any cat can become affected (Giugliano et al., 2024; and Viste et al., 2002). Approximately 83–85% of feline mammary gland tumours are malignant, with carcinoma being the most common tumour type (Simeonov, 2024; and Srisawat et al., 2024). The clinical stage, including tumour size, lymph node involvement, and metastatic dissemination, has been shown to be associated with survival (Petrucci et al., 2021, Viste et al., 2002; and Weijer & Hart, 1983). Therefore, early tumour detection by routine mammary gland palpation, prompt diagnosis, and rapid institution of treatment are essential. Radical mastectomy with or without adjuvant chemotherapy is considered the treatment of choice for malignant tumours (Giménez et al., 2010) as it has been shown to reduce the risk of recurrence when compared to simple lumpectomy or regional mastectomy (MacEwen et al., 1984). For benign tumours, however, the less extensive surgical options may be sufficient (Giménez et al., 2010).

Fine-needle aspiration cytology is a simple, quick, non-invasive, and cost-effective method to help determine the origin and malignancy of a mass-like lesion (Dolka et , 2018). However, it is based on the evaluation of single cells and cell clusters and cannot determine the relationship between the tumour cells and those of the underlying tissue, so histopathology is required for definitive diagnosis (Giménez et al., 2010). Core-needle biopsies provide an alternative to fine-needle aspiration biopsies and can yield a larger sample but are also associated with a higher risk of complications such as haemorrhage (O'Connor et al., 2002). Both methods are commonly used to diagnose breast cancer in humans (Verma et al., 2021), whereas in dogs, their diagnostic accuracy is compromised by the high prevalence of complex and mixed tumours (Sontas et al., 2012). In cats, most mammary gland tumours are composed of one epithelial cell population (Sammarco et al., 2020) which can improve the diagnostic accuracy of cytology, but until recently, peer-reviewed scientific literature related to the topic has been scarce.

After a comprehensive literature search, three scientific articles (Pakdeesaneha et al., 2024, Simeonov, 2024; and Amorim et al., 2006) addressing the PICO question were identified. One (Amorim et al., 2006) is a descriptive case series in which the diagnostic accuracy of cytology was reported along with other data about the clinical, cytological, and histopathological findings, clinical staging, treatment, and outcome of patients with mammary gland masses. Being a descriptive study, it is particularly prone to selection bias and lacks a control group which reduces its internal and external validity. Therefore, it provides weak evidence. The other two (Pakdeesaneha et al., 2024; and Simeonov, 2024), however, are diagnostic accuracy studies that were specifically designed to compare the diagnostic accuracy of cytology to histopathology. Therefore, they have the potential to provide strong evidence. However, all the studies are flawed by multiple important limitations which reduce the overall quality of evidence. Firstly, the sample size was small in two of the studies (Pakdeesaneha et al., 2024; and Amorim et al., 2006), and in the third one (Simeonov, 2024), only the number of tumours was reported instead of actual patients. None of the studies provided a sample size calculation or other explanation on how the sample size was determined. These factors reduce the reliability of the results as random variation can have a major effect on the collected data. Secondly, geographical bias may be present in all the studies as each of them was performed at a single geographic location. Thirdly, two of the studies (Pakdeesaneha et al., 2024; and Amorim et al., 2006) were undertaken in referral practice which may have introduced referral bias. These factors further limit the generalisability of the results.

Two of the papers (Pakdeesaneha et al., 2024; and Simeonov, 2024) did not describe the study population in sufficient detail, and in two of the papers (Simeonov, 2024; and Amorim et al., 2006), no inclusion or exclusion criteria were stated. This reduces the external validity of the studies. In contrast, the only study which did include the inclusion and exclusion criteria (Pakdeesaneha et al., 2024) may have been affected by selection bias as identifying eligible patients on the basis of planned mastectomy can lead to overrepresentation of patients with malignant tumours. This may bias the results towards diagnostic accuracy of cytology as malignant tumours with cells from one single origin may be easier to accurately diagnose by cytology than other masses (Pakdeesaneha et al., 2024). Additionally, the number of benign tumours was not clearly stated by the authors but based on Table 2, it can be calculated to be 2/64 (3%). This percentage is considerably lower than the reported prevalence (15–16.7%) of naturally occurring benign mammary gland tumours in cats (Simeonov, 2024; and Srisawat et al., 2024). This factor further reduces the external validity of the study.

The materials and methods were inadequately described in all of the studies. In the study by Amorim et al. (2006), the sampling and sample preparation techniques, the number and experience of the investigators preparing and evaluating the samples and the number of cytological malignancy criteria required to be met for classifying the tumour as malignant were not reported.  In the study by Simeonov (2024), the number of the collected cytological samples, the active ingredients of the fixative and stain, the number and collection method of the histopathological samples and the number and experience of the investigators collecting and evaluating the samples were not reported. In addition, old editions of cytology and pathology books (Goldschmidt et al., 2017; Misdorp et al., 2001; and Tyler et al., 1999) were used as the source of applied malignancy criteria instead of a recommended grading system such as the Elston and Ellis system, also known as Nottingham Histological Grade (Elston & Ellis, 1991), which was originally developed for human breast cancer but has been applied to feline mammary tumours for more than 20 years (Avallone et al., 2021; and Cassali et al., 2020), or the Mills grading system, which was specifically designed for feline mammary carcinoma (Mills et al., 2015). Not using a recommended grading system may add more subjectivity to the malignancy assessment.

In the study by Pakdeesaneha et al. (2024), only two or three cytological samples were collected from each tumour, and the distance between the cytological and histopathological sampling site was not reported which can introduce measurement bias and falsely reduce the reported diagnostic accuracy of cytology. On the other hand, the cytological samples were obtained from anaesthetised patients which differs from common practice and reduces the generalisability of the results as sampling under general anaesthesia may improve the visibility and immobility of the target tissue and improve the diagnostic quality of the collected samples when compared to ones collected from conscious or mildly sedated patients. This could bias the results towards diagnostic accuracy of cytology. In contrast, histopathology was performed on a small excisional biopsy instead of the entire surgically removed mammary gland tissue which could potentially reduce the diagnostic accuracy of histopathology used as the clinical reference standard. Furthermore, whether a certified veterinary pathologist evaluated the cytological samples was not clearly stated even though the importance of the pathologist’s experience was emphasised in the introduction and can have an effect on the reliability of the results. The same holds true for the two other studies (Simeonov, 2024; and Amorim et al., 2006) in which the experience of neither the investigator of the cytological or histopathological samples was stated. These key aspects may impact the reliability of the results, and the inadequate reporting hampers the critical assessment of the internal validity of the studies and reduces their reproducibility. In addition, the described differences in sample collection, preparation, evaluation, and grading represent a potential source of interobserver and between-study variation in the diagnostic accuracy of cytology, although in this case, the observed differences were small.

There is also some ambiguity regarding the design of the studies. In one of the papers (Amorim et al., 2006), the study type was not stated at all whereas in another one (Simeonov, 2024), the temporal design (i.e. whether prospective data collection was planned before sample collection or whether previously collected samples were retrospectively retrieved for data collection) was unclear. As retrospective studies may be more prone to selection bias, missing important data and collecting data of lower quality than prospective studies (Talari & Goyal, 2020), not providing this piece of information substantially compromises the critical assessment of the study’s internal validity. Additionally, the study’s apparent lack of blinding can introduce performance bias as being aware of the results of either the cytological or histopathological assessment can subconsciously guide the researcher’s interpretation of the other modality’s results and lead to a falsely high agreement between the two. In contrast, the authors of Pakdeesaneha et al. (2024) did report the use of blinding in the histopathological evaluation process, as well as randomisation in the allocation of patients to undergo core-needle biopsies with different-sized needles. However, neither the blinding nor the randomisation process weredescribed in sufficient detail to allow critical assessment of their validity. In addition, whether indeterminate results were present and how they were handled was not reported in any of the studies, even though this might have had a considerable effect on the detected diagnostic accuracy of cytology, especially considering the small sample sizes.

As for reporting the results, two of the papers (Pakdeesaneha et al., 2024; and Amorim et al., 2006) did not include the sensitivity, specificity, positive predictive value or negative predictive value of cytology, and confidence intervals were not stated in any of the papers. Two studies (Simeonov, 2024; and Amorim et al., 2006) failed to discuss their limitations, sources of potential bias and generalisability of the results. In addition, the incidence of adverse effects related to the cytological and histopathological sampling was not reported in any of the papers, not even the one utilising core-needle biopsies and discussing their potential risks (Pakdeesaneha et al., 2024).

Despite the abovementioned limitations, all three studies reached the same conclusion that fine-needle aspiration cytology is a highly accurate diagnostic method to predict the malignancy of feline mammary gland tumours with its overall sensitivity (either reported or calculated by the author of this Knowledge Summary) ranging from 93% to 100%. However, only one of the studies (Simeonov, 2024) included a large enough number of benign tumours (n = 18, 15%) to allow a meaningful specificity calculation according to which the overall specificity was lower (75%) than the sensitivity (95.2%).

In terms of the utility of cytology in clinical practice, it is of utmost importance that no malignant tumours are missed and therefore left undertreated or untreated. The high sensitivity of cytology demonstrated in all the three studies translates to a low, but non-negligible risk of missing a malignancy. Therefore, any lumpectomies or regional mastectomies of cytologically diagnosed benign tumours should be followed by a histopathological examination of the entire excised tissue, so that in the case of a preoperative misdiagnosis, a more aggressive approach can be adopted. Another option would be to try to confirm the benign nature of a tumour preoperatively by an incisional biopsy, but especially in the case of a small mass, this may prove less feasible than a simple excision of the entire tumour.

In contrast, misdiagnosing benign tumours as malignant can lead to other types of unfortunate consequences, such as unnecessarily invasive surgical procedures or unjustified euthanasia decisions. Therefore, it would be important to gain more information about the specificity of cytology in predicting the malignancy of feline mammary gland tumours. However, the low incidence of benign tumours makes it challenging to collect a sufficient sample size, as demonstrated by the three studies (Pakdeesaneha et al., 2024, Simeonov, 2024; and Amorim et al., 2006). To overcome this problem, further carefully planned large-scale studies, possibly with a multicentre approach, are warranted. In the meantime, in cases in which the owner is leaning towards euthanasia rather than an invasive surgical procedure, confirmation of the cytological diagnosis via an incisional biopsy should be considered.

In conclusion, these three studies (Pakdeesaneha et al., 2024, Simeonov, 2024; and Amorim et al., 2006) provide moderate evidence to show that cytology is an accurate diagnostic method for predicting the malignancy of feline mammary gland tumours when compared to histopathology as the clinical reference standard, at least in terms of sensitivity. In addition, one of the studies (Pakdeesaneha et al., 2024) provides weak evidence to suggest that the diagnostic accuracy of core-needle biopsies is nonsuperior to fine-needle aspiration biopsies. However, further carefully planned studies with larger sample sizes, especially in terms of benign tumours, are required to confirm the findings and to gain more information about the specificity of cytology. Due to the current uncertainties, all preoperative cytological diagnoses should be confirmed with a postoperative histopathological examination.

## Methodology

**Search Strategy:** 

Databases searched and dates covered:	CAB Abstracts on CABI Digital Library platform 1973–Week 2 2025PubMed via the NCBI website 1970–Week 2 2025Web of Science 1974–Week 2 2025Scopus 1978–Week 2 2025
Search strategy:	CAB Abstracts:(cat* OR feline*) AND ((mammary AND) tum*r OR neoplas* OR *carcinoma*) AND (cytolog* OR fine*needle))PubMed:(cat OR cats OR feline*) AND (mammary) AND (cytolog* OR fine*needle)Web of Science:(cat or cat or feline or felines) and mammary and (tumor or tumour or neoplasm or neoplasia) and (cytology or cytological or fine-needle or fine needle)Scopus:(cat or cat or feline or felines) and mammary and (tumor or tumour or neoplasm or neoplasia) and (cytology or cytological or fine-needle or fine needle)
Dates searches performed:	11 January 2025

**Exclusion / Inclusion Criteria:** 

Exclusion:	Studies irrelevant to the PICO question (e.g. studies investigating other feline tumours, studies investigating mammary gland tumours in other species than cats and studies investigating other aspects of feline mammary gland tumours than the diagnostic accuracy of cytology). Case reports and other studies with only one relevant case. Duplicated papers. Papers retracted due to unguaranteed scientific integrity. Non-peer reviewed literature (e.g. conference abstracts and book chapters). Non-English language papers.
Inclusion:	Studies relevant to the PICO question, involving more than one relevant case and published in English.

**Search Outcome:** 

Database	Number of results	Excluded – papers irrelevant to the PICO question	Excluded – papers with only one relevant case	Excluded – duplicated papers	Excluded – retracted papers	Excluded – non-peer-reviewed literature	Excluded – non-English language papers	Total relevant papers
CAB Abstracts	104	67	11	1	0	19	4	2
PubMed	113	107	2	1	1	0	0	2
Web of Science	32	24	3	1	1	1	0	2
Scopus	23	13	2	0	2	4	0	2
Total relevant papers when duplicates removed								3

## ORCiD

Juulia Virtanen: https://orcid.org/0009-0004-9711-9127

## Conflict of Interest

The authors declare no conflicts of interest.

## Disclaimer

Knowledge Summaries are a peer-reviewed article type which aims to answer a clinical question based on the best available current evidence. It does not override the responsibility of the practitioner. Informed decisions should be made by considering such factors as individual clinical expertise and judgement along with patient’s circumstances and owners’ values. Knowledge Summaries are a resource to help inform and any opinions expressed within the Knowledge Summaries are the author's own and do not necessarily reflect the view of the RCVS Knowledge. Authors are responsible for the accuracy of the content. While the Editor and Publisher believe that all content herein are in accord with current recommendations and practice at the time of publication, they accept no legal responsibility for any errors or omissions, and make no warranty, express or implied, with respect to material contained within. For further information please refer to our Terms of Use.
